# A New Model of the Mechanisms behind Glucose Oxidase Action in Plant Insect Interactions

**DOI:** 10.1007/s10886-025-01648-5

**Published:** 2025-10-15

**Authors:** Jared Griffin, Sahil Pawar, Gary W. Felton

**Affiliations:** Department of Entomology, Pennsylvania State University, State College, PA USA

**Keywords:** Glucose oxidase, Plant-insect interactions, Elicitor, Effector, Induced defenses

## Abstract

Plants and insect herbivores are in a constant co-evolutionary arms race. Plants are always under the threat of insect herbivory and need to employ defenses against insect herbivores, which in turn employ counter defense strategies. The salivary enzyme glucose oxidase (GOX), found in many caterpillar species, has been documented to attenuate defenses in plants such as *Nicotiana tabacum* (cultivated tobacco). However, in *Solanum lycopersicum* (cultivated tomato), glucose oxidase elicits defensive responses. Multiple mechanisms have been proposed for how GOX affects plant signaling, but there is still considerable disagreement about which is correct. In this review, we review existing models on the mode of GOX action and propose a new model to fill in research gaps and better explain the mechanism behind GOX action. Our model, coined the “ROS Threshold-Dependent Defense Toggle Model”, proposes that whether a plant activates jasmonic acid or salicylic acid-mediated defenses depends on the amount and persistence of hydrogen peroxide whose levels are dependent upon ROS-scavenging capabilities of the plant. We also emphasize the use of cultivated tomato as a model system to test our proposed model.

## Introduction

Plants are constantly under threat of insect herbivory. Being sessile in nature, they rely on physical defenses (such as trichomes) that are often complemented by chemical defenses (secondary metabolites such as alkaloids and phenolics) to deter insect herbivores (Kariyat et al. 2018; Paudel et al. 2019; Musser et al. 2002). Chemical defenses can be constitutive (always present in the plant) or induced (produced in response to an insect attack) and often act as a second line of defense for plants. Induced defenses are often regulated by plant signaling molecules and/or phytohormones such as jasmonic acid (JA) and salicylic acid (SA). Chewing herbivory causes an accumulation of JA in the attacked plant (McCloud and Baldwin 1997; Karssemeijer et al. 2020), while SA accumulation takes place upon feeding by piercing-sucking or phloem-feeding insects (Li et al. 2006; Thaler et al. 2010). These phytohormones induce plant signaling pathways that activate plant defenses and deter insect feeding. It is worth noting that SA and JA show an antagonism where SA or its derivatives counteract JA and its effects on defense signaling (Doherty et al. 1988; Pena-Cortes et al. 1993; Doares et al. 1995). Once plants upregulate their defenses, insects adapt by evolving counter defense strategies. These include behavioral changes in which insects avoid feeding on defense-induced tissue (Hulley 1988; Dussourd 1999; Dussourd and Denno 1991), or chemical/physiological means of countering plant defenses such as metabolizing and detoxifying toxic plant-derived compounds (Synder et al. 1993; Snyder et al. 1994) or interfering with plant defense by using the contents of saliva to suppress plant defenses (Acevedo et al. 2019).

Some molecules have been discovered in insect oral secretions like volicitin, which induces maize volatiles that attract parasitoids (Turlings et al. 2000), or inceptin, which triggers increases in JA and SA (Schmelz et al. 2006). Among salivary enzymes, glucose oxidase (GOX) has attracted attention due to its contradictory effects across plant species. GOX from Lepidopteran caterpillars can both induce (Tian et al. 2012; Lin et al. 2020) and suppress (Musser et al. 2002; Musser et al. 2005; Zong and Wang 2004; Deizel et al. 2009; Bede et al. 2006) plant defenses. In the present snapshot of this evolutionary arms race between plants and insects, it seems difficult to generalize the role of GOX in plant-insect interactions, whether it acts as an elicitor of plant defenses or suppressor. Furthermore, there is disagreement concerning its primary mode of action, as this changes with the specific system of study. Despite numerous proposed mechanisms for the mode of action of GOX, there is no clear consensus on which mechanism is correct. Discovering the precise mechanism is critical for understanding the role of GOX in plant-insect interactions. Our discussion aims to review the current state of GOX in plant-insect interactions, propose a new model of GOX action, and examine potential strategies in using tomatoes as a system to elucidate the mechanism of GOX action.

## Glucose Oxidase

Glucose oxidase (GOX) belongs to the GMC oxidoreductase family (Cavener 1992) and was initially discovered in the fungal species *Aspergillus niger* (Heller and Ulstrup 2021). In insects, GOX was initially discovered in the caterpillar *Helicoverpa zea* in 1999, and since then has also been reported in many other Lepidopteran caterpillars (Eichenseer et al. 1999, 2010). Although primarily observed in caterpillar saliva, it is also expressed in caterpillar labial glands (that produce saliva), caterpillar midguts, and other tissues. It catalyzes the production of hydrogen peroxide (H_2_O_2_) and d-gluconolactone from glucose in the presence of oxygen (Cavener 1992). GOX has also been found in bees and honey, where it acts as an antimicrobial agent (White et al. 1963), plays a role in detoxifying plant nectar by reducing phenolic content (Liu et al. 2005), and has been proposed to contribute to social immunity (Lopez-Uribe et al. 2017).

Compounds derived from insects (such as insect oral secretions or insect saliva) can be broken down into two functional categories: effectors (which disrupt/suppress plant defensive responses) and elicitors (which elicit/induce plant defensive responses) (Jones et al. 2022). GOX has been reported to play both roles. GOX from *H. zea* was initially reported as an effector in the cultivated tobacco plant *Nicotiana tabacum*, reducing the amount of nicotine (a plant defense) accumulated in leaves (Musser et al. 2002). Feeding by two other GOX-producing *Helicoverpa* species: *H. armigera* and *H.assulta* suppressed nicotine induction in *N. tabacum* compared to mechanical wounding (Zong and Wang 2004). Application of *H. zea* labial gland extract with active GOX to mechanically damaged *N. tabacum* leaves also showed suppression of nicotine accumulation compared to wounded controls (Musser et al. 2005). A study on *Medicago truncatula* showed that fungal GOX and H_2_O_2_ application to mechanically damaged leaves resulted in lower transcription of certain defense-related genes (Bede et al. 2006).Generalist caterpillars typically have higher amounts of GOX compared tospecialist caterpillars (Eichenseer et al. 2010), possibly linked to a generalists’ need to suppress defenses in a wider variety of plants (although later research on other plant systems calls into question how widespread GOX’s benefits as an effector are). GOX has also been shown to cause stomatal closure in tomatoes (Lin et al. 2021), soybean (Lin et al. 2021), and maize (Jones et al. 2023), which might reduce plant volatile signaling, and also inhibits certain green leaf volatiles while enhancing the release of terpene volatiles in maize (Jones et al. 2023; Gao et al. 2024) acting as a suppressor of certain indirect plant defenses.

In cultivated tomato however, multiple studies have consistently documented the fact that GOX acts as an elicitor of plant defenses. Trypsin inhibitor levels were significantly higher in wounded tomato plants compared to non-wounded tomato plants, and insects feeding showed a two- fold reduction in weight on wounded tomato plants (Musser et al. 2005). Salivary gland extracts with active GOX showed lower trypsin inhibitor levels than plants treated with purified GOX, water, or inactivated GOX salivary extracts (Musser et al. 2005). Caterpillar saliva is released through an opening in the caterpillar’s lip called the spinneret, which is external to the mouth cavity. Cauterization of the spinneret prevents secretion of saliva from labial glands. A study on *Arabidopsis thaliana* found that cauterizing *Spodoptera exigua* caterpillar spinnerets increased JA levels at least 4 times more than those seen in plants exposed to caterpillars with intact spinnerets (Weech et al. 2008), indicating the importance of saliva-derived compounds in plant defense manipulation. *H. zea* caterpillars with intact spinnerets significantly induced higher *PIN2* gene expression (a JA-inducible gene that is involved in the synthesis of proteinase inhibitors) than caterpillars with ablated spinnerets in tomatoes after 48 h (Tian et al. 2012). The same study reported that JA levels were around 13 and 40 times higher in saliva-treated plants than in buffer-treated and control plants respectively after 4 h of treatment (Tian et al. 2012). These findings indicate that saliva induces early JA induction while defense genes are induced much later. However, our unpublished data indicates that GOX is not responsible for the JA elicitation. In another study, applying mechanical damage and fungal GOX to cultivated tomato plants led to a high level of trypsin inhibitor when compared to other treatments (Lin et al. 2020).

With GOX playing dual roles of defense suppression and induction, there have been multiple proposals for how GOX causes these differences in plant defenses (Table 1). The exact mechanism of GOX action however, remains undecided.

**Table 1:** Proposed mechanisms of GOX action

Hypothesis	Summary	Strengths	Weaknesses
JA-SA Antaonism (Musser et al. 2002)	GOX interferes with the JA pathway by activating the SA pathway.	Supported by several studies in which SA or its derivatives disrupt JA or its related defensive products (Doares et al.^1995^; Doherty et al. 1988; Pena-Cortes et al. 1993).	Disagrees with a follow-up study in which SA- deficient tobacco plants still had a reaction to GOX (Musser et al. 2005).
GOX as Negative Ethylene Regulator (Diezel et al. 2009)	GOX negatively regulates ethylene production and ultimately leads on an increased SA response.	Agrees with other work that suggests ET is modulator o SA-JA interaction (Leon-Reyes et al. 2009) and other studies that report a synergy between ET an methyl jasmonate (Penninckx et al. 1998; Xu et al. 1994).	Does not account for previous work that shows that GOX suppresses defenses even in SA-deficient tobacco (Musser et al. 2005), or why certain plants have an induced response to GOX (Lin et al. 2020; Tian et al. 2012). Additionally, it is puzzling what caterpillars with high GOX would gain from inducing SA production if other studies indicate that caterpillars with low GOX production can decrease nicotine production (Kahl et al. 2000; Winz and Baldwin 2001).
Ethylene Nicotine Inhibition (Zong and Wang 2004)	GOX produces hydrogen peroxide, which produces ET that inhibits nicotine biosynthesis.	Agrees with reports that ethephon (releases ET) when added to plants at the same time as methyl jasmonate leads to a reduction in leaf nicotine induction (Winz and Baldwin 2001) and work that shows that application of caterpillar oral secretions reduced nicotine concentration (Kahl et al. 2000).	Does not address why SA deficient plants have response to GOX (Musser et al. 2005) or why another study showed caterpillars with high GOX production to act as negative regulators of ET (Diezel at al. 2009).
Hydrogen Peroxide as Secondary Messenger (Orozco-Cardenas et al. 2001)	Hydrogen acts as a secondary messenger for the JA pathway and only activates late defensive genes.	Explains why cultivated tomato has an induced response to GOX. Agrees with later work that shows induced reponses of tomato cultivars to GOX (Lin et al. 2020; Tian et al. 2012).	Does not explain why GOX acts as an effector in certain plant systems (Lin et al. 2020; Tian et al. 2012).
ROS Scavenging (Lin et al. 2020)	Differences in ROS scavenging explain why plant species react differently to GOX.	Attempts to explain why certain plants system have different responses to GOX. Agrees with the ideas of hydrogen peroxide as a secondary messenger for the JA pathway (Orozco-Cardenas et al. 2001).	Does not address studies in which high hydrogen peroxide concentrations lead to SA induction (Leon et al. 1995; Neuenschwander et al. 1995; Summermatter et al. 1995).
JA_SA Antagonism and ROS Scavenging (Yang et al. 2023)	GOX produces hydrogen peroxide, which activates the SA pathway to ineterfere with JA signaling while peroxidase scavenges hydrogen peroxide and activate defenses.	Integrates JA-SA antagonism and ROS scavenging ideas.	Does not address how hydrogen peroxide seems to act as secondary messenger for the JA pathway (Orozco-Cardenas et al. 2001).
GOX Receptor (Tian et al. 2012)	Plants have a receptor for GOX akin to how plants recognize pathogens.	Agrees with plant pathology literature on R genes (Parker et al. 2000).	No GOX receptors have been found, and hydrogen peroxide can account for many effects without the need for specific GOX receptors (Orozco-Cardenas et al. 2001).
Hydrogen Peroxide Receptors/Sensors ( current paper)	Hydrogen peroxide sensors/receptors may a role in the plants response to GOX since they have demonstrated roles in plants signaling.	Recent findings implicate hydrogen peroxide sensors in several areas signaling (Bi et al. 2022; Wu et al. 2020).	Specific pathways that would relate to the GOX response have not been mapped out to our knowledge.

* The table was made in Rstudio (Posit team  2025) using the packages tidyverse (Wickham et al. 2019), kableExtra (Zhu 2024), and gt (Iannone et al. 2025)

### Mechanisms of GOX Action

#### Phytohormone Signaling

One proposed mechanism of GOX action is through the activation of phytohormonal crosstalk. In the publication that first reported GOX as an effector, the authors speculated that GOX may be inhibiting JA or disrupting it by interfering in its interactions with other phytohormonal pathways (Musser et al. 2002). While this hypothesis is supported by previous studies that demonstrate JA-SA antagonism, it was not supported by their follow up study: on transgenic, SA-deficient tobacco plants, caterpillars feeding with intact salivary glands elicited lower amount of nicotine induction than caterpillars feeding with ablated salivary glands, which led the authors to speculate that this was a change not solely mediated by SA and that perhaps ethylene biosynthesis was involved in GOX’s suppression of nicotine (Musser et al. 2005). A study on *Nicotiana attenuata* found that herbivory by *Spodoptera exigua* (caterpillar with higher GOX activity) resulted in higher SA levels and lower JA levels than *Manduca sexta* herbivory (Diezel et al. 2009). In a study done prior to the discovery of GOX in caterpillars, it was discovered that *H. zea* feeding resulted in a significant increase of SA in *Gossypium hirsutum* (cotton) (Bi et al. 1997 ).These results are further complicated by studies on other Solanaceous plant species such as tomatoes that show elevated JA-mediated defenses in response to GOX (Tian et al. 2012; Lin et al. 2020). Therefore, there seems to be species-specificity in GOX interaction with SA and JA.

Additionally, supplementing *M. sexta* oral secretions with GOX decreased ethylene (ET) emissions by 25% compared to *M. sexta* oral secretions alone, suggesting a modulation to the ET pathway (Diezel et al. 2009). They postulated that GOX may act as a negative regulator of ET production in *N. attenuata* which could affect other phytohormone levels (Diezel et al. 2009). A prior study hypothesized that GOX leads to H_2_O_2_ production, which then leads to ET production that results in the inhibition of nicotine biosynthesis (Zong and Wang 2004). The Diezel model agrees with other work that suggests ET is a modulator of SA-JA interactions (Leon-Reyes et al. 2009) and other studies that report a synergy between ET and methyl jasmonate (Penninckx et al. 1998; Xu et al. 1994). However, it does not account for previous work that shows that GOX suppresses defenses even in SA-deficient tobacco (Musser et al. 2005), or why certain plants have an induced response to GOX (Lin et al. 2020; Tian et al. 2012). Additionally, it is puzzling what caterpillars with high GOX would gain from inducing SA production if other studies indicate that caterpillars with low GOX production can decrease nicotine production (Kahl et al. 2000; Winz and Baldwin 2001). The Zong and Wang hypothesis agrees with reports that ethephon (releases ET) when added to plants at the same time as methyl jasmonate leads to a reduction in leaf nicotine induction (Winz and Baldwin 2001) and work that shows that application of caterpillar oral secretions reduced nicotine concentration (Kahl et al. 2000), but does not address why SA-deficient plants have a response to GOX (Musser et al. 2005) or why a future study showed caterpillars with high GOX production to act as negative regulators of ET (Diezel et al. 2009). Taken together, these studies imply that there is more behind GOX action than simply activating a single phytohormonal pathway using JA, SA, or ET. GOX may activate different phytohormonal pathways in different plant species, however the mechanism(s) underlying this interaction are unclear.

#### Hydrogen Peroxide as Secondary Messenger

Another proposed mode of action for GOX is through the production of reactive oxygen species (ROS), namely, hydrogen peroxide (H_2_O_2_), which can act as a secondary messenger downstream of the JA-phytohormonal pathway One such model suggests H_2_O_2_ acts as a secondary messenger in tomato plants to activate late defensive genes (Orozco-Cardenas et al. 2001). Plant leaves contain substantial levels of glucose, which serves as the substrate for GOX and results in H_2_O_2_ production. It has been demonstrated that excised tomato leaves supplemented with glucose and treated with GOX activated plant defense genes, but not signaling genes (Orozco-Cardenas et al. 2001). Applying H_2_O_2_ has been able to elicit similar defensive responses to GOX in some plant species (Lin et al. 2020). This model is also supported by other studies that show H_2_O_2_ plays a role in plant signaling (Bi et al. 2022; Wu et al. 2020). These studies indicate that the presence of GOX-derived H_2_O_2_ elicits plant responses and plays a role in plant signaling. Different plants can contain different levels of glucose, resulting in varied H_2_O_2_ production, which can partly explain species-specific differences in GOX action. This model only explains GOX-induction of tomato defenses, without explaining why other plant species react differently to GOX.

#### ROS Scavenging Efficiency

This hypothesis partially explains species-specific responses through antioxidant capacity of plants. Several *Solanaceous* plants showed varying reactions (either an induction of defensive proteins or no response at all relative to other treatments) to GOX treatment (Lin et al. 2020). Some plants do not show a response to GOX treatment: In sorghum, (*Sorghum bicolor*) GOX treatment was unable to suppress the expression of sorghum flavonoid pathway genes (Shinde et al. 2024). While tomatoes and soybeans showed GOX-induced stomatal closure, cotton plants were unaffected (Lin et al. 2021). The authors of these papers suggested that differences in plant responses could be due to how effectively plants can scavenge ROS, causing variations in defensive responses and stomatal dynamics (Lin et al. 2020, 2021). This hypothesis attempts to explain why there are different reactions to GOX but does not acknowledge studies in which H_2_O_2_ leads to SA activation (Leon et al. 1995; Neuenschwander et al. 1995; Summermatter et al. 1995). A recent publication presented a model in which the herbivore *Plutella xylostella* (diamondback moth) uses H_2_O_2_ to activate the SA pathway and interfere with JA signaling while peroxidase is used to scavenge H_2_O_2_ and activate defenses (Yang et al. 2023). The authors also note that the differences in plant response may be due to differences in ROS scavenging between plant species (Yang et al. 2023). This hypothesis addresses JA-SA antagonism, but does not directly address H_2_O_2_ as a secondary messenger for the JA pathway (Orozco-Cardenas et al. 2001). Overall, it seems likely that improper ROS scavenging can result in the continuous presence of ROS molecules resulting in varied signaling responses.

#### GOX and Hydrogen Peroxide Receptors/Sensors

This hypothesis suggests direct molecular recognition and a dedicated pathway towards GOX action. Initial speculations of the presence of a possible GOX receptor in plants akin to R-gene-like receptors for pathogens have been made (Tian et al. 2012), with a recent review referring to this idea too (Kallure et al. 2022). However, no GOX receptors have been identified yet, and most phenomena can be explained by H_2_O_2_-mediated responses. The idea of a receptor is based upon the study of plant pathology (Parker et al. 2000) and therefore is possible, but to our knowledge, no researchers have directly searched for a GOX receptor. This may be due to the large focus on H_2_O_2_ which plays a demonstrated role in plant defense signaling that may not necessitate a GOX receptor. The presence of a GOX receptor and further molecules in a signaling cascade would prove more towards a specialized pathway dedicated to GOX action, rather than GOX action via shared molecules with other signaling pathways.

While there are no reported GOX receptors, there have been many reports of H_2_O_2_ receptors, which still leave open the possibility that GOX responses are still partly receptor mediated. H_2_O_2_ receptors and sensors in *A. thaliana* have been investigated (Bi et al. 2022; Wu et al. 2020). Theleucine-rich-repeat receptor kinase HPA1C was found to be activated by H_2_O_2_. HPA1C is required for stomatal closure by mediating H_2_O_2_ induced activation of calcium channels in guard cells (Wu et al. 2020). The cytosolic thiol peroxidase PRXIIB was found to act as a sensor for H_2_O_2_, playing a role in stomatal immunity (Bi et al. 2022). These H_2_O_2_ sensors have an established role in plant signaling but could also be involved in plant responses to GOX. Other researchers found that GOX caused stomatal closure in the cultivated tomato and *Glycine max* (soybean), but not in *Gossypium hirsutum* (cotton) (Lin et al. 2021). As these receptors and sensors influence stomatal dynamics, it seems that variations in them may partially explain why plants have different responses to GOX. However, the receptors are most likely part of a more complex response including crosstalk with phytohormonal pathways and to our knowledge no receptors have been specifically linked to GOX action.

While all these mechanisms have been proposed, none have been definitively and widely demonstrated (Table 1). GOX signaling has varying results. Plants within the same family seem to react differently to GOX despite their somewhat close relation (Musser et al. 2005; Lin et al. 2020). Additionally, some of the hypotheses mentioned appear to be restricted to specific systems and are not widely generalizable. The question then becomes why are these plants reacting differently to GOX, and which mechanism, if any, is correct?

## GOX Regulation by External Factors

GOX activity and expression are influenced by diverse abiotic and biotic factors which can lead to context-dependent functional outcomes. Elevated temperatures alter carbohydrate metabolism pathways linked to GOX functionality. A study showed that *H. zea* caterpillars reared on lower temperatures showed higher amounts of salivary GOX, potentially because of a growth-immunity trade-off. Caterpillars reared at lower temperatures also elicited higher amounts of defense proteins in tomatoes post herbivory (Paudel et al. 2020). Plant secondary metabolites and sugar content shape GOX activity in herbivores. For example, *H. armigera* feeding on artificial diet (high sugar, no defenses) exhibited elevated GOX activity compared to those feeding on tobacco leaves (Hu et al. 2008). In another study, *H. zea* larvae feeding on cotton and soybean showed lower GOX activity than diet-fed or geranium-fed *H. zea* larvae (Eichenseer et al. 2010). This shows how GOX activities are affected by insect diet. A recent study has shown that certain bacterial strains from diamondback moth gut regurgitant decreased the expression of GOX genes (Qiao et al. 2024). Finally, bacteria and polydnaviruses (PDVs) can also affect the secretion of GOX (Tan et al. 2018; Wang et al. 2017b). *H. zea* caterpillars infected with PDVs from parasitoid wasps reduced GOX transcript levels and dampened plant immune responses, which led to enhanced caterpillar growth rates (Tan et al. 2018). The fact that PDVs had such an effect on GOX is notable, as there are an estimated 17,000–46,000 + wasps in the subfamily Microgastrinae, which are Braconid wasps that have symbioses with bracoviruses (a type of polydnavirus) (Rodriguez et al. 2012). Polydnaviruses also affect ichneumonid wasps (Tanaka et al. 2007), so the number of effected wasps is even larger. The large number of wasp species that carry polydnaviruses may mean that caterpillars are frequently having their ability to use GOX impaired in the field. However, since GOX has a range of effects this may not always be detrimental to the caterpillar. This multi-trophic interaction between a virus, parasitoid, caterpillar, and plant further stresses on how multiple factors could lead to differential effects across systems.

It is also important to note that GOX does not exist and function in isolation. Insects have multiple other salivary constituents that could function in influencing GOX activity. Local glucose concentrations can be increased by activities of other salivary enzymes such as salivary amylase (hydrolyzes starch) (Asadi et al. 2010; Rivera-Vega et al. 2017), fructosidase (cleaves sucrose into glucose and fructose) (Rivera-Vega et al. 2017), and glucosidases (hydrolyze polysaccharides into glucose) (Rivera-Vega et al. 2017). Increased glucose (substrate) availability for GOX can enhance GOX activity. It has been demonstrated that insects can sequester plant carotenoids in many places of the body, including the mandibular and labial salivary glands (Eichenseer et al. 2002). These carotenoids may act as antioxidants, but the authors of the previous study pointed out that the antioxidant effects of the carotenoids were not enough to protect insects from the prooxidant properties of tomato leaves. These components can create a dynamic system influencing GOX action.

## A New Model for GOX Action: ROS Threshold Dependent Defense Toggle

To address the limitations of previous models of GOX action, we propose a new model. Briefly, the reaction of GOX with glucose results in the production of H_2_O_2,_ which acts as a signaling molecule. Species-specific antioxidant enzyme pathways determine H_2_O_2_ persistence in plants. H_2_O_2_-concentration dependent phytohormonal pathway activation leads to the activation of plant defense genes. Low H_2_O_2_ concentrations activate JA while high concentrations result in SA dominance.

Plant defensive signaling is influenced by many factors. The Orozco-Cardenas model suggests that when a plant is damaged, the phytohormone JA mediates the activation of early defense genes and H_2_O_2_ acts as a secondary messenger to activate late defense genes (Orozco-Cardenas et al. 2001). Their model decouples phytohormones from certain aspects of defense signaling, since theoretically H_2_O_2_ alone should be enough to activate defensive genes. Furthermore, since one of the products of GOX action is H_2_O_2_, it is possible that H_2_O_2_ production alone is sufficient to explain the elicitation of plant defenses. Their study also agrees with the findings of two other models both of which report elicitation of defenses for the cultivated tomato (Tian et al. 2012; Lin et al. 2020).

However, it conflicts with Musser’s first hypothesis (Musser et al. 2002) and the Diezel model (Diezel et al. 2009). Although both the Diezel model (Diezel et al. 2009) and the Orozco-Cardenas model (Orozco-Cardenas et al. 2001) claim H_2_O_2_ as the responsible molecule for triggering defenses related to phytohormonal pathways, the Diezel model claims it is responsible for activating the SA pathway while Orozco-Cardenas claim that it activates defensive genes that are related to the JA pathway (although Diezel and colleagues also say that GOX may be activating SA directly). It is possible that the reaction to GOX is species specific, but this still does not explain the underlying mechanism of what’s driving the observed differences.

The Orozco-Cardenas model (Orozco-Cardenas et al. 2001) and the Diezel model (Diezel et al. 2009) also come into conflict with the GOX receptor hypothesis. Again, if H_2_O_2_ is responsible for the defensive response, then a plant would not necessarily require a GOX-specific receptor to mount a defensive response and would benefit from the evolution of a specialized H_2_O_2_ pathway, independent of GOX action. This idea coupled with the fact that no GOX receptor has been identified make the GOX receptor model currently unsupported (although to our knowledge, no one has actively searched for such a receptor). If H_2_O_2_ is what leads to defense signaling, and it is implemented in both antagonistic pathways, then what explains the difference observed in how different species react to GOX?

Another more serious difficulty is that there is a body of literature that shows that H_2_O_2_ causes a secondary ROS burst, (comprising of multiple other ROS molecules) which leads to the activation of the SA pathway. One study found that injecting certain concentrations of H_2_O_2_ into cultivated tobacco caused significant increases in SA levels (Leon et al. 1995). Another study that did similar work on *A. thaliana* found that by injecting leaves with H_2_O_2_ concentrations of 50 mM or higher caused changes in the amounts of bound SA (free SA increased during the first 24 h, and then SA concentrations returned to basal levels which was interpreted as a conversion to the bound form of SA) (Summermatter et al. 1995). How can plants be using the same molecule, i.e. H_2_O_2_, to activate both pathways? This is partly addressed by the reactive oxygen species scavenger hypothesis (Lin et al. 2020) and a recent model (Yang et al. 2023).

Insect herbivory or the presence of insect-derived compounds can trigger rapid bursts of reactive oxygen species (ROS) in plants within minutes (Block et al. 2018; Sperdouli et al. 2022). The production of ROS such as H_2_O_2_ and superoxide (O^2−^) is often mediated by membrane-localized oxidases. These ROS molecules participate in signaling cascades that activate various defense responses. They can act as secondary messengers, being involved in changes in gene expression and inducing the production of defensive compounds (Orozco-Cardenas et al. 2001). ROS molecules are also involved in many hormonal pathways such as the JA and SA pathway which further regulate plant defense responses (Orozco-Cardenas et al. 2001; Leon et al. 1995; Louis et al. 2013) and are even involved in auxin signaling (Roy et al. 2025).

To manage the potentially damaging effects of excessive ROS, plants have evolved sophisticated ROS-scavenging mechanisms. These include enzymatic antioxidants such as catalase, peroxidase, and superoxide dismutase, as well as non-enzymatic antioxidants like ascorbic acid, glutathione, phenolics, carotenoids, and non-protein thiols (Prasad et al. 1994; Li and Yi 2012; Yu et al. 2019b; Shen et al. 2018). The delicate balance between ROS production and scavenging is crucial towards maintaining cellular redox homeostasis and determining the outcome of ROS signaling (Bi and Felton 1995).

Some plants seem to be able to metabolize ROS relatively quickly, as applications of exogenous H_2_O_2_ at concentrations as high as 10 mM to soybean cell suspension cultures were eliminated within 10 min, indicating that perhaps H_2_O_2_ effects are dose dependent (Levine et al. 1994). This agrees with other observations that only certain concentrations caused significant increases in SA levels (Leon et al. 1995) as well as another study that showed that out of three concentrations of H_2_O_2_ tested, only the highest concentration caused free SA accumulation that differed from the control in *N. tabacum* (Neuenschwander et al. 1995). It is also supported by a recent paper that demonstrated that systemic acquired resistance and the associated accumulation of SA in *A. thaliana* is dependent upon an optimal dose of H_2_O_2_ (Cao et al. 2024). This would mean that ROS scavenging enzymes are equally relevant as ROS molecules themselves, as they decide how quickly a species can deal with the accumulation of ROS.

**Fig. 1:**
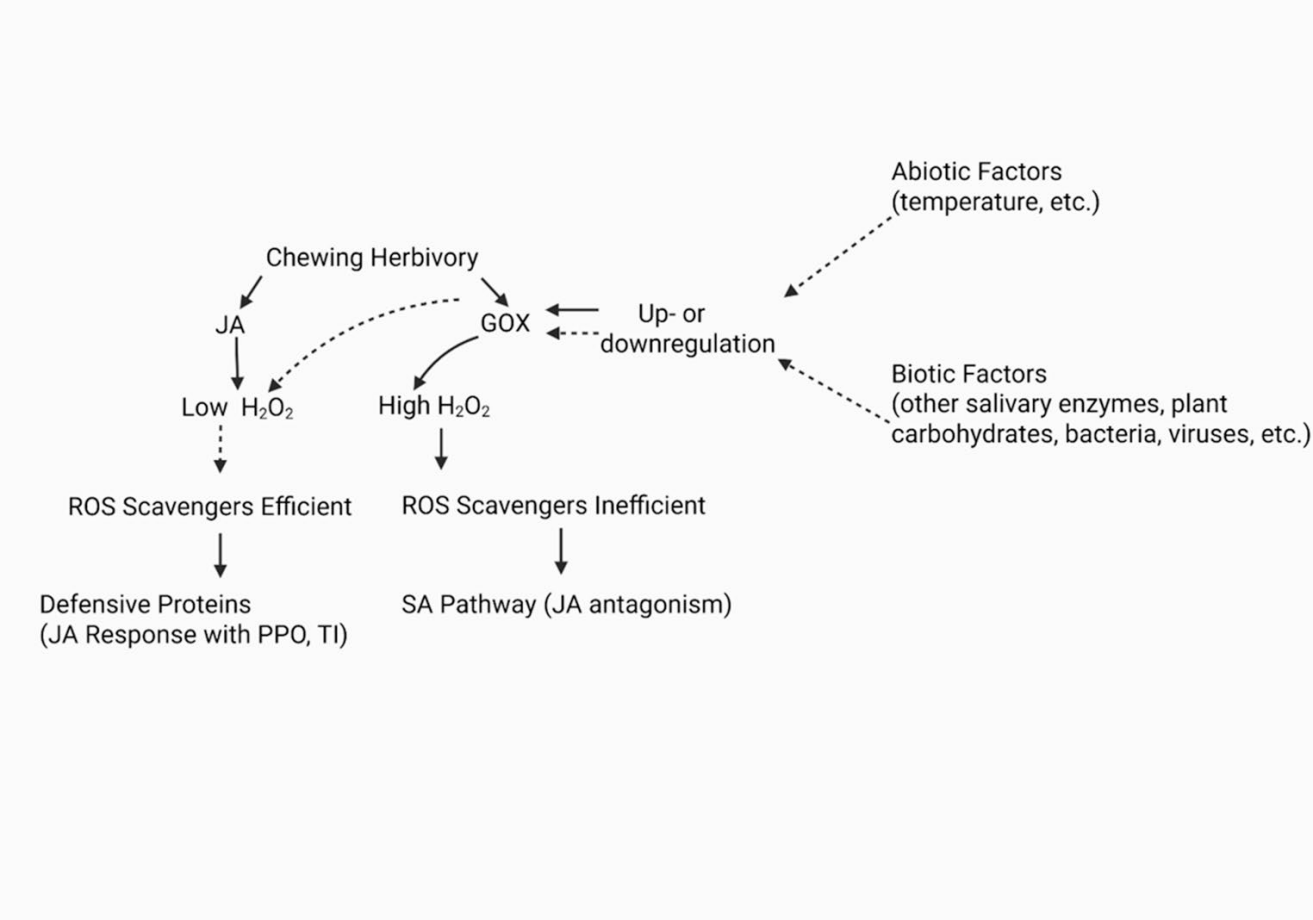
A new proposed model of GOX action. The act of herbivory and the secretion of GOX both create an accumulation of hydrogen peroxide, while GOX itself is influenced by abiotic and biotic factors (which also influences the amount of hydrogen peroxide produced). If the ROS scavengers are efficient, the late defensive genes related to the JA pathway are activated (which produce defensive responses like polyphenol oxidase and protease inhibitors). If the ROS scavengers are inefficient, the SA pathway is activated and this causes JA antagonism. Created in BioRender. Griffin, J. (2025) https://BioRender.com/q43r809

Our new model states that both the act of herbivory and subsequent exposure to GOX create ROS bursts (H_2_O_2_), that induce phytohormonal signaling. Which defense pathway is activated and maintained (SA or JA pathway) depends upon the concentration and rate of H_2_O_2_ production as well as the efficiency of the ROS scavenging enzymes (Fig. 1). GOX may act as an elicitor when H_2_O_2_ is kept under a certain concentration and may act as an effector when it hits a higher concentration and elicits an SA burst. Under our model H_2_O_2_ acts as a secondary messenger of the JA pathway, as in the Orozco-Cardenas model (Orozco-Cardenas et al. 2001). The concept of a threshold for H_2_O_2_ is based upon several studies in which SA concentrations are only significantly increased after certain concentrations of H_2_O_2_ are applied to plants (Leon et al. 1995; Summermatter et al. 1995; Neuenschwander et al. 1995). While these studies may be used for a general suggestion of relevant H_2_O_2_ concentrations, we predict that the amount of H_2_O_2_ needed to switch from a JA response to an SA response may vary based on the focal leaf, age of the plant, and method/amount of H_2_O_2_ application. We suggest exposing plants to a broad range of H_2_O_2_ concentrations and mapping the phytohormonal/defensive response for multiple plant species and multiple leaves within the same plant species to discover specific thresholds.

It is important to note that these predictions apply to a locally damaged leaf. Systemic signaling in plants is variable and not a part of our model. To our knowledge, only one study has investigated systemic leaf signaling in the context of the effects of GOX (Lin et al. 2020).A recent study demonstrated that H_2_O_2_ sulfenylates a transcription factor (CHE - CCA1 Hiking Expedition) only in systemic leaves which leads to Systemic Acquired Resistance (SAR), while higher levels of ROS in local tissues cause further oxidation of cysteine residues in CHE (Cao et al. 2024). This study leaves open the possibility that insects that produce high levels of GOX may be able to cause systemic signaling in certain plants, which could be extremely costly for a plant to do if it is dealing with pests that are vulnerable to JA-related defenses and instead produces SA-related defenses.

It is also important to note that plant responses may be different in different leaves within the same plant due to factors relating to age such as differences in ROS scavenging ability. This is an important distinction, as many previous studies do not seem to highlight the differences that may be apparent in GOX-induced signaling within a single plant. Other additional factors may include the availability of substrate (in this case, glucose), as the availability of glucose determines the production of hydrogen peroxide. It was previously demonstrated that defense gene expression was only induced by a combination of GOX and glucose, not by GOX alone (Orozco-Cardenas et al. 2001). Another study found that by supplementing mechanical damage treatments in maize with GOX and glucose, they could cause significantly higher induction of maize protease inhibitor compared to mechanical damage treatments involving GOX and glucose alone (Louis et al. 2013). Other researchers also noted in their work that the differences they observed between species in response to GOX may be due to differences in plant carbohydrates such as glucose, starch, and fructose content (e.g., starch may be broken down into glucose by salivary amylase) (Lin et al. 2020). Together, these results indicate that important carbohydrate differences in plants may contribute heavily to how a plant reacts to GOX.

Finally, while we use the term concentration regarding H_2_O_2_ it is also likely that lower concentrations at higher amounts may cause results similar or identical to those seen using smaller amounts of H_2_O_2_ at higher concentrations. While this model does seem to resolve some difficulties that other models do not, it requires rigorous testing. One system that is attractive for research is the cultivated tomato.

## Cultivated Tomato as a System To Investigate GOX Action

The cultivated tomato is a Solanaceous plant related to other crops such as eggplants, peppers, and potatoes. Originating from Mesoamerica (Razifard et al. 2020; Blanca et al. 2022), it has since become a globally produced vegetable with 186 million tons being produced in 2022 (FAO 2023). Besides being an economically important crop, it has served as a model system for many avenues of research like plant pathology, stress tolerance, genetics, and plant-insect interactions. Multiple abiotic factors such as extreme temperatures (Dat et al. 1998; Prasad et al. 1994), high intensity light (Dat et al. 2003; Vandenabeele et al. 2004) and drought (Moran et al. 1994; Selote and Khanna-Chopra 2004) have the potential to produce oxidative stress/ROS accumulation in plants which could lead to damage. For this reason, topics like these have been studied in tomato with the goal of identifying resistant genotypes. Several studies exist comparing the heat tolerance (Abdul-Baki 1991 ; Haque et al. 2021), drought tolerance (Conti et al. 2019; Zhou et al. 2017), chilling tolerance (Cao et al. 2015), and salt tolerance (Pawar et al. 2025) of different types of tomatoes. The tomato genome was sequenced in 2012 (The Tomato Genome Consortium 2012). This has led to projects such as an examination of the genetic variation in multiple tomato accessions and wild relatives (The 100 Tomato Genome Sequencing Consortium et al. 2014) and genetic examinations of tomato evolution (Lin et al. 2014).

Tomatoes have been widely utilized to understand plant-insect interactions. The plant enzyme polyphenol oxidase (PPO) was demonstrated as an anti-nutritive defense in tomato (Felton et al. 1989), with further investigations looking into proteinase inhibitors (Glassmire et al. 2024; Lin et al. 2020; Paudel et al. 2019). The well-mapped out defensive responses of tomato make it an ideal system to scrutinize the mechanism of GOX. Taken together, tomato is a system that has been studied intensively. It is a model system for biotic and abiotic stressors. Its genome has been sequenced for over a decade, and it has been used extensively in plant-insect interactions. Specific tools that could be used in this system include: genome-edited lines, ROS reporters, and JA/SA pathway mutants. Several gene-edited lines of tomatoes have been used to investigate abiotic stressors that involve H_2_O_2_ (Yu et al. 2019a, ; Wang et al. 2017a, ). Cultivated tobacco (a relative of tomato) has previously used the transgenes as ROS reporters (Mur et al. 2005), and recent work has developed two green fluorescent protein (GFP)-based ROS reporters for *A. thaliana* (Lim et al. 2019) which may be able to be adapted to the tomato system given its well-researched nature. NahG (SA deficient) tomato plants have been used to investigate stress (Jansma et al. 2022) and pathogen resistance (Brading et al. 2000). Furthermore, the elicitation of plant defenses in this system via GOX makes it a desirable system to use when dissecting the mechanisms that GOX acts through.

We propose a variety of methods of investigation to use to test the proposed model. Since the model relies heavily upon hydrogen peroxide persistence and ROS-scavenging efficiency, a direct way to test the model is to quantify the concentration of hydrogen peroxide in plants that have different reactions to GOX (induced and suppressed responses) such as in the cultivated tomato and cotton (or *N. attenuata*). This could be accomplished by using feeding assays with insects (such as *H. zea*) or by mechanical damage with repeated applications of hydrogen peroxide. This should be coupled with investigations of the gene expression of ROS scavengers, as we predict that species/genotypes with more efficient scavenging will have a JA-pathway related response to GOX. Another method of investigation would be to investigate whether a plant such as the cultivated tomato responds to GOX with defense suppression if the ROS scavenging enzymes it has are impaired (this could be done using knockout plants, including multi-gene knockouts of several important ROS scavengers and by inducible gene silencing to investigate the complex network of redox systems). A final suggestion would be to see if improved ROS scavenging efficiency would lead to the opposite (a defense induction in a system where GOX usually causes suppression). While these proposals are possible, there are technical challenges that are involved. Measuring precise ROS concentrations is difficult, and isolating the effects of GOX is complicated by the redundancy in the antioxidant enzyme network. If our proposed model proves to be incorrect, this may be due to non-linear accumulation of local signals (such as biphasic increases in H_2_O_2_) or complex interactions between ROS and other signaling pathways that have so far not been adequately explored in the context of GOX research.

## Conclusion and Future Directions

GOX is at the convergence of many areas of research such as plant-insect interactions, ecology, physiology etc. It is an enzyme that has been studied in many plant systems, with species-specific effects and unclear mechanisms of action. Although many models of GOX action have been proposed, none seem definitive. In this review, we propose a new model which aims to resolve many difficulties posed by previous models but needs further rigorous testing to validate.

The model system of tomato plants offers many advantages to investigate the mechanisms of GOX. Mainly, GOX has been shown to induce defenses in tomato, unlike other systems where GOX suppresses defenses. If the model proposed in this paper is correct, overcoming the H_2_O_2_ threshold should be able to push tomatoes from showing defense elicitation to defense suppression under GOX.

Although this review has largely focused on the molecular mechanisms behind GOX, there are many other avenues of research still open to investigation. GOX is a widespread enzyme in Lepidoptera, and therefore it may play an important role in plant-insect interactions in the wild. Much of the knowledge accumulated about GOX comes from cultivated species (Musser et al. 2002; Musser et al. 2005; Tian et al. 2012; Lin et al. 2020; Lin et al. 2021). It would be useful to observe the reactions of wild plants to this enzyme to ascertain the ecological role it plays in plant-insect interactions. The sharply contrasting roles of GOX as an elicitor and an effector make it possible that it plays an important role in choosing host plants. There are many wild species of plants like tomato and tobacco available that make these investigations feasible.

GOX may be an important part of several ecological interactions. As generalist Lepidoptera generally produce more GOX than specialists (Eichenseer et al. 2010), GOX likely is more relevant for caterpillars that come into contact with a wide range of plants. While it is currently hard to generalize the effect that GOX has on plants, it may allow caterpillars to deal with a wide variety of plant defenses by suppressing plant defenses. It would be valuable to correlate the host range of an insect that secretes a large amount of GOX like *H. zea* to whether the host plant has a defensive response to GOX or not. Some Lepidopterans have been reported to somehow sense chemical defenses in plants before oviposition such as *Danaus plexippus* (the monarch butterfly) as they oviposit on *Asclepias curassavica* (tropical milkweed) plants with intermediate cardenolide concentrations (Agrawal et al. 2021), so a study like this would indicate whether GOX may play a role in the selection of a host plant by ovipositing moths. Relatedly, GOX may also contribute to the feeding behaviors of caterpillars. It has been reported that *H. zea* caterpillars with intact spinnerets induce higher densities of type VI glandular trichomes in *Solanum lycopersicum* cv. MicroTom tomatoes compared to caterpillars with ablated spinnerets (Tian et al. 2012). This could potentially affect how caterpillars behaviorally deal with feeding if this phenomenon is widespread. It would be informative to investigate whether any behavioral adaptations arise due to this, and this could be achieved by feeding observations.

In line with the ecological role that GOX may play, it may also offer insights into fields like induced defense theory. There has been a considerable amount of research on induced defenses (Lin et al. 2020; Paudel et al. 2019; Agrawal et al. 2014) and many papers on the theory behind their benefit and evolution (Agrawal 2005; Karban et al. 1997; Karban and Myers 1989). Authors have predicted that induced defenses are favored in environments with variable herbivory (Adler and Karban 1994) however measuring the costs of induced defenses has proven difficult (Brown 1988; Karban 1993). GOX research would add to our understanding of induced defenses because it modulates plant defenses. Studies that don’t appreciate this may be either over or underestimating the true range of induced defenses that a plant is capable of. This is especially pertinent because of the range of GOX in insects such as Lepidoptera (Eichenseer et al. 2010). Much of the earlier writing on the theory behind induced plant resistance focuses on plants. There does not seem to be as much attention paid to the complexity of the herbivore side of the interactions in terms of elicitors and effectors as many of the older papers predate the discovery of molecules like GOX.

In this review, we have proposed a new model for the mechanism of GOX action. Our new model builds upon previous work in several ways. While other previous authors have noted that ROS scavenging is likely important (Lin et al. 2020; Yang et al. 2023), we have made an important distinction. Oxidative damage can be harmful to plants (Larkindale 2002), making ROS scavenging a vitally important function. In our model, however, inefficient ROS scavenging instead leads to SA pathway activation. This is consistent with the observations of several studies that SA activation is dependent upon certain concentrations of H_2_O_2_ (Leon et al. 1995; Neuenschwander et al. 1995). We have also drawn attention to the fact that GOX may cause different signaling responses within a plant, since ROS scavenging may differ between leaves due to age. Several papers document differences in antioxidant and/or ROS scavenging abilities and/or transcripts between younger and older leaves (Casano et al. 1994; Cspregi et al. 2017; Dertinger et al. 2003; Li et al. 1995; Moustaka et al. 2015). It is also important to note that while we focus on SA pathway activation, SA activation and oxidative damage are not necessarily mutually exclusive. There is evidence that SA can inhibit ROS scavenging enzymes and increase hydrogen peroxide concentrations (Chen et al. 1993). However, it is important to note that one study reported that even at a concentration of 1mM, SA was not enough to inhibit cotton foliar ROS scavengers like catalase in vitro, so there may be a level of system specificity in this regard as well (Bi et al. 1997). We have also taken systemic signaling into consideration, which to our knowledge only one study on GOX has addressed (Lin et al. 2020).

Another aspect of GOX research that has been largely neglected is the potential of GOX’s other product, gluconolactone. Although the paper that established GOX as an effector mentioned that gluconic acid itself when applied to leaf wounds caused a 29.3% reduction in nicotine content compared to a control consisting of damage and water (Musser et al. 2002), other studies have focused almost solely on the role of H_2_O_2_ in defense signaling. Gluconolactone is converted spontaneously into gluconic acid (Bentley and Neuberger 1949), and this may lead to a change in pH. This is important, as if too much accumulates there is a chance that this product of GOX may destabilize the enzyme (although the disruption in pH may also affect the functioning of plant proteins involved in defense responses such as PPO and ROS scavengers). More research should be conducted in this vein, as this aspect of possible interference is very pertinent to how long GOX may be effective in altering plant defense signaling. It is also important to note that certain microbes possess lactonases, which break down lactones (Brodie and Lipmann 1955). It would be informative to know if symbiotic plant microbes can disrupt GOX action through breaking down gluconolactone into gluconic acid. Finally, although GOX likely plays an important role in plant-insect interactions it is only one piece of the puzzle. There are many other elicitors and effectors that insect herbivores possess. Likewise, there are many aspects of plant defense signaling that are still poorly understood. Studying these interactions will help us to begin to understand the rich complexity of the evolutionary arms race between plants and insects. We close with several points that still need to be addressed by research:

- What is the quantitative H_2_O_2_ threshold for switching between JA and SA responses?
- If the quantitative H_2_O_2_ threshold does exist, how much does it vary between species? Between different leaves on the same plant?
- How does GOX interact with other insect salivary components?
- What roles does GOX play in shaping insect feeding preferences, behaviors, or host selection in natural ecosystems?
- Are there specific receptors for GOX-derived H_2_O_2_?
- Do gluconolactone and gluconic acid play larger roles than are recognized?
- Do specific GOX receptors exist?

## Data Availability

No datasets were generated or analysed during the current study.
